# Supplementary material for: Genome-Wide Identification of the TGA Gene Family and Expression Analysis under Drought Stress in Brassica napus L
Source: Int J Mol Sci. 2024 Jun 8;25(12):6355. doi: 10.3390/ijms25126355 (PMC11203523; doi:10.3390/ijms25126355)
Supplement: Supplementary file 1 [file ijms-25-06355-s001.zip › ijms-3023940-supplementary.pdf]

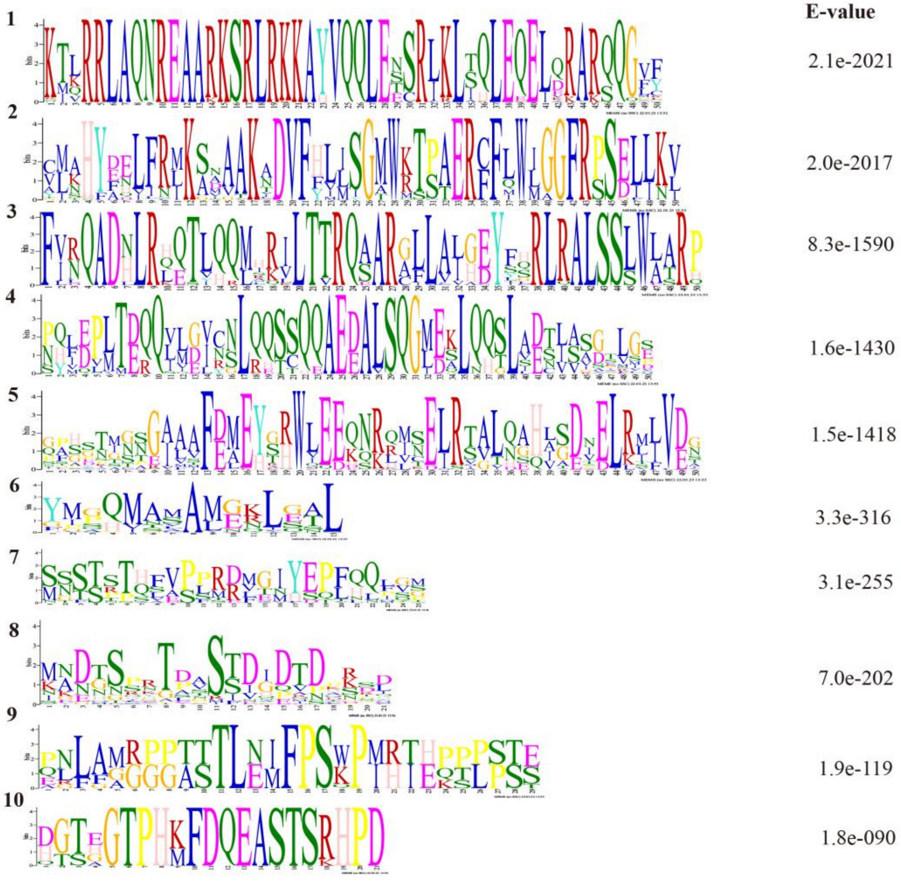

Figure S1. Sequence logos of 10 motifs in *Brassica napus* TGA proteins.

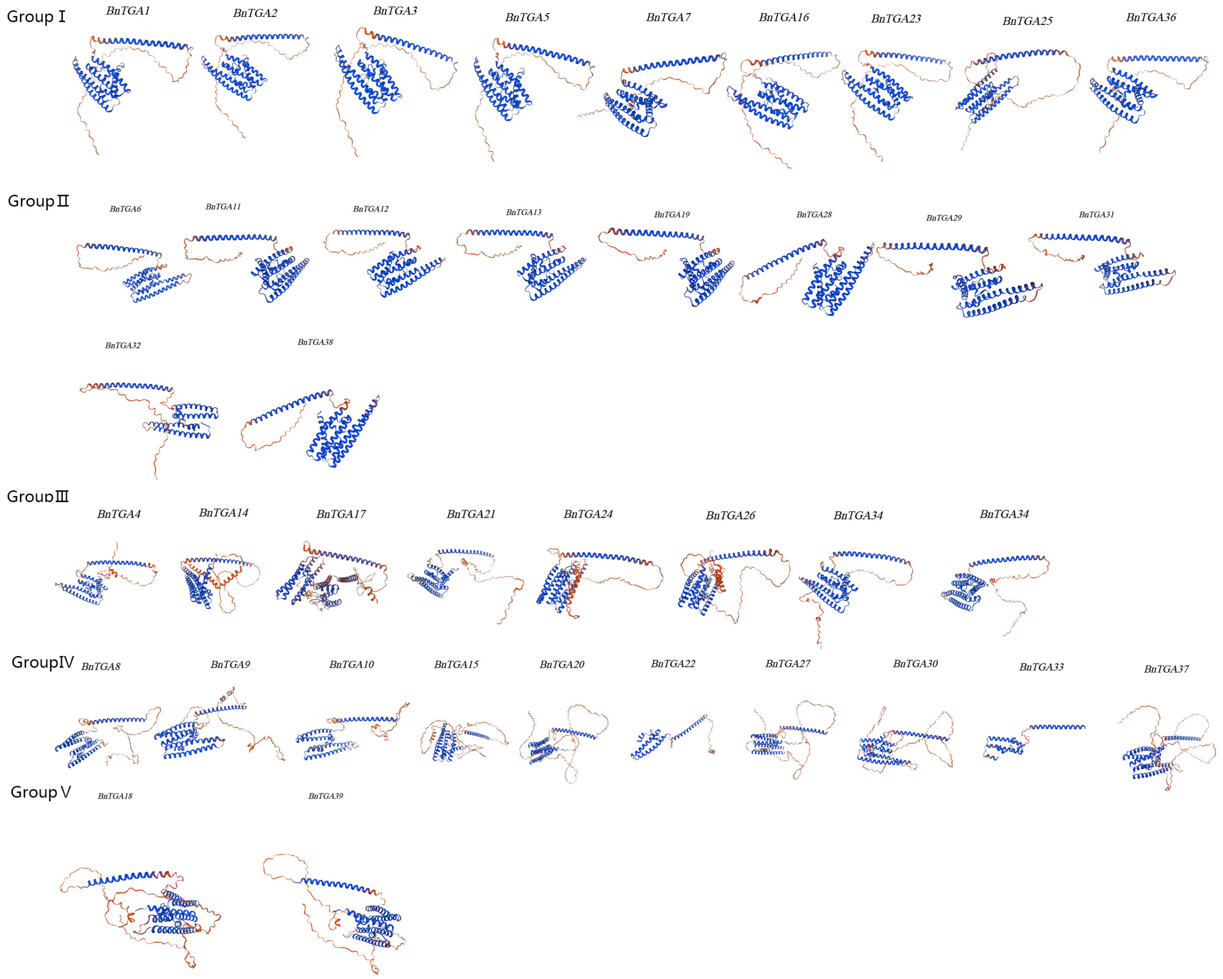

Figure S2. The predicted three-dimensional (3D) structure of BnTGA proteins.

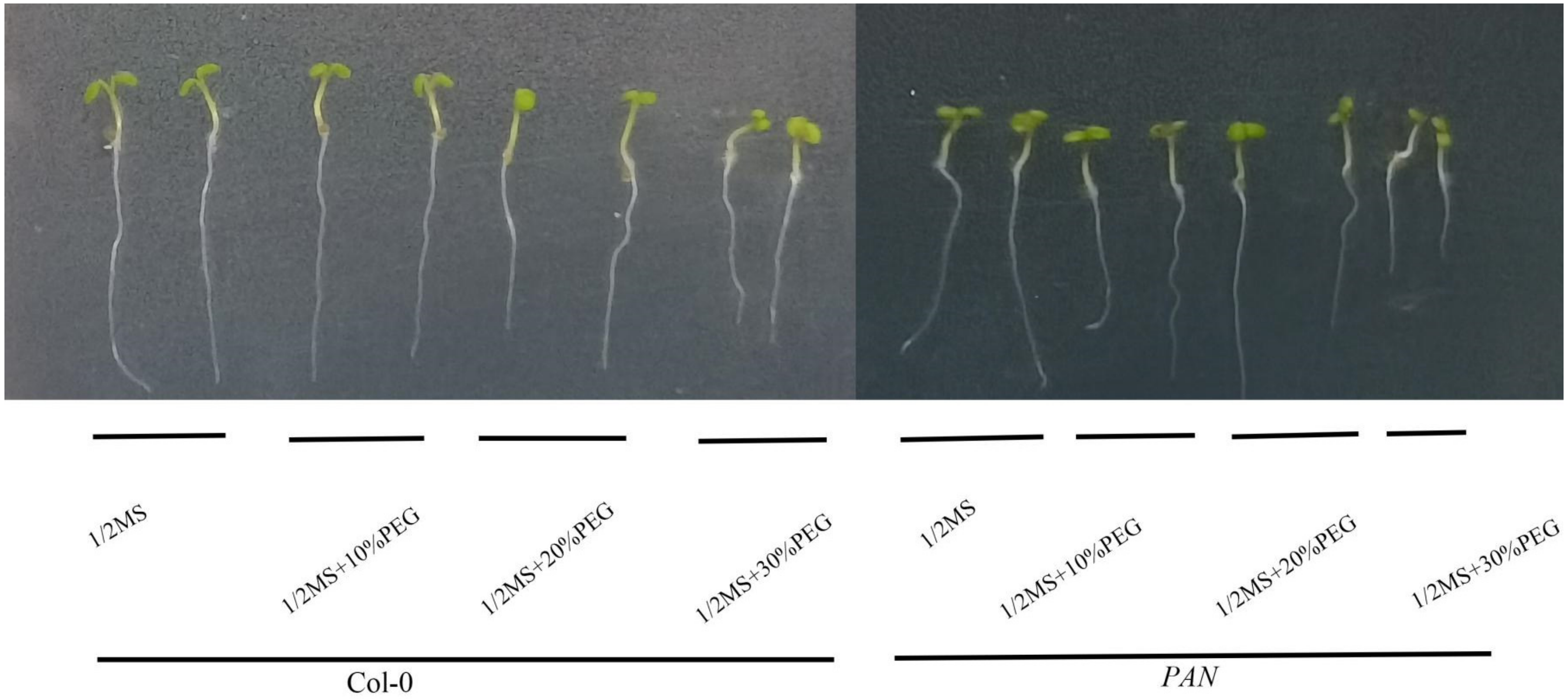

Figure S3. *Arabidopsis thaliana* wild type and *PAN* mutant were grown on 1/2MS medium for 7 days.

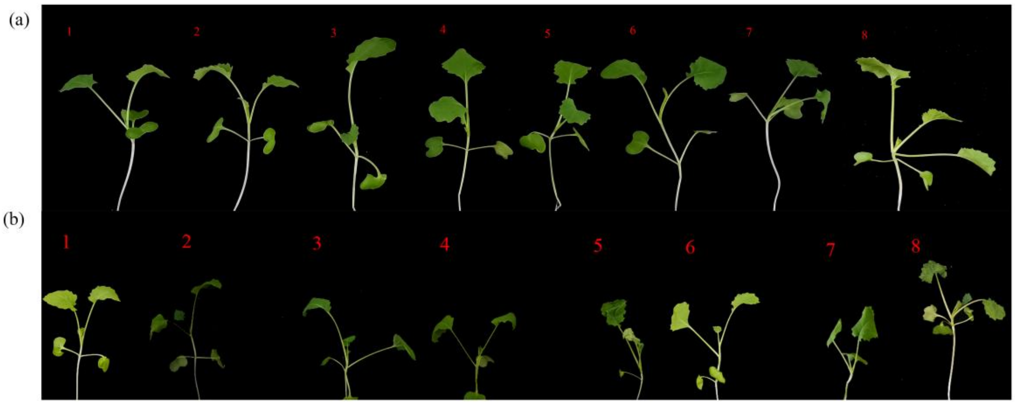

Figure S4. (a) Phenotypic diagram of experimental materials before drought treatment. (b) Phenotypic diagram of experimental materials after drought treatment. Marks 1 – 8 are LY8, WJY520, LY07, LY1008H, DY6, ZS11, LY31AB, and ZD630, respectively.

Table S1 The genomic and biochemical information for *TGA* genes identified in *B.napus*.

gene ID	Gene name	protein length (aa)	MW (Da)	pI	LOC	Chromosomal location
BnaAnng04720D	BnTGA1	370	42211.84	7.8	nucl: 14	chrAnn_random
BnaA06g24140D	BnTGA2	367	41951.46	7.3	nucl: 13.5	chrA06
BnaC02g43620D	BnTGA3	370	42225.83	7.81	nucl: 14	chrC02
BnaA07g20770D	BnTGA4	371	41986.06	6.08	nucl: 14	chrA07
BnaC09g46670D	BnTGA5	364	41805.36	6.43	nucl: 13.5	chrC09
BnaC02g01780D	BnTGA6	326	36643.06	8.87	nucl: 14	chrC02
BnaC02g00560D	BnTGA7	362	41686.19	6.88	nucl: 13.5	chrC02
BnaCnng67350D	BnTGA8	364	40462.91	6.4	nucl: 13	chrCnn_random
BnaC08g43230D	BnTGA9	428	47527.78	8.68	nucl: 14	chrC08
BnaUnng03970D	BnTGA10	352	39461.72	7.06	nucl: 14	chrUnn_random
BnaAnng01920D	BnTGA11	326	36643.06	8.87	nucl: 14	chrAnn_random
BnaA05g26790D	BnTGA12	331	36851.37	8.66	nucl: 14	chrA05
BnaC03g37460D	BnTGA13	331	36757.26	8.99	nucl: 14	chrC03
BnaC06g38430D	BnTGA14	430	49302.7	6.52	nucl: 14	chrC06
BnaA09g48940D	BnTGA15	484	53915.7	7.02	nucl: 14	chrA09
BnaC03g49070D	BnTGA16	367	42012.61	8.33	nucl: 13.5	chrC03
BnaC06g20630D	BnTGA17	570	65513.34	5.85	nucl: 14	chrC06
BnaA07g27320D	BnTGA18	442	49777.86	5.72	nucl: 13	chrA07
BnaA10g24020D	BnTGA19	327	36377.13	8.92	nucl: 14	chrA10
BnaA06g04770D	BnTGA20	478	53327.14	7.28	nucl: 13.5	chrA06
BnaC08g20170D	BnTGA21	370	41682.93	6.77	nucl: 14	chrC08
BnaAnng36310D	BnTGA22	198	22873.3	9	nucl: 14	chrAnn_random
BnaA10g22150D	BnTGA23	364	41817.27	6.61	nucl: 13.5	chrA10

BnaA09g30910D	BnTGA24	418	47703.66	6.07	nucl: 14	chrA09
BnaC09g06840D	BnTGA25	389	44422.32	8.28	nucl: 14	chrC09
BnaA07g33790D	BnTGA26	444	51065.86	7.25	nucl: 14	chrA07
BnaC05g06030D	BnTGA27	478	53234.97	7.28	nucl: 13.5	chrC05
BnaA03g32170D	BnTGA28	331	36830.37	8.99	nucl: 14	chrA03
BnaC09g48640D	BnTGA29	328	36487.22	8.92	nucl: 14	chrC09
BnaA10g24100D	BnTGA30	451	50181.51	6.72	nucl: 13.5	chrA10
BnaA01g30760D	BnTGA31	334	37122.79	7.89	nucl: 14	chrA01
BnaC05g40810D	BnTGA32	324	36050.33	5.84	nucl: 14	chrC05
BnaAnng33510D	BnTGA33	209	24463.68	9.36	nucl: 12	chrAnn_random
BnaC05g17700D	BnTGA34	385	43531.66	5.82	nucl: 14	chrC05
BnaA08g20970D	BnTGA35	369	41750.8	6.53	nucl: 14	chrA08
BnaA02g00310D	BnTGA36	362	41615.15	7.22	nucl: 13.5	chrA02
BnaC09g48720D	BnTGA37	451	50177.53	6.75	nucl: 13.5	chrC09
BnaCnng22950D	BnTGA38	333	37047.7	7.89	nucl: 14	chrCnn_random
BnaC06g30310D	BnTGA39	442	49765.87	5.77	nucl: 13	chrC06

Table S2 Prediction analysis of secondary structure of proteins encoded by *TGA* gene families in *B.napus*

Gene number	Alpha helix(%)	Beta turn(%)	Random coil(%)	Extended strand(%)
BnTGA1	60.00	2.16	31.08	6.76
BnTGA2	59.95	1.91	32.15	5.99
BnTGA3	58.65	2.70	32.97	5.68
BnTGA4	61.19	1.89	31.54	5.39
BnTGA5	62.91	2.75	28.02	6.32
BnTGA6	68.71	1.84	25.46	3.99
BnTGA7	61.88	2.21	28.18	7.73
BnTGA8	52.75	2.20	37.91	7.14
BnTGA9	52.57	3.04	36.45	7.94
BnTGA10	57.67	2.56	31.82	7.95
BnTGA11	67.79	2.45	23.93	5.83
BnTGA12	75.23	2.11	17.22	5.44
BnTGA13	68.88	2.11	23.87	5.14
BnTGA14	62.09	2.56	31.16	4.19
BnTGA15	50.00	2.69	41.12	6.20
BnTGA16	62.13	2.18	29.70	5.99
BnTGA17	55.79	4.56	31.93	7.72
BnTGA18	50.45	2.71	34.62	12.22
BnTGA19	68.20	1.53	24.77	5.50
BnTGA20	48.74	3.14	41.21	6.90
BnTGA21	60.81	3.24	30.27	5.68
BnTGA22	58.08	2.02	30.30	9.60
BnTGA23	62.64	2.47	28.85	6.04
BnTGA24	62.20	2.87	27.75	7.18
BnTGA25	59.64	2.83	31.88	5.66
BnTGA26	60.36	2.48	31.98	5.18
BnTGA27	50.42	2.09	40.79	6.69
BnTGA28	66.16	1.81	24.77	7.25
BnTGA29	65.85	2.13	27.13	4.88
BnTGA30	52.33	1.55	41.24	4.88
BnTGA31	66.47	1.80	25.45	6.29
BnTGA32	59.26	2.78	28.09	9.88
BnTGA33	63.64	3.83	20.10	12.44
BnTGA34	58.70	2.60	31.43	7.27
BnTGA35	60.98	2.17	30.89	5.96
BnTGA36	63.26	2.49	27.07	7.18
BnTGA37	51.66	2.66	40.13	5.54
BnTGA38	66.37	2.10	25.83	5.71
BnTGA39	55.20	2.71	31.45	10.63

Table S3 The germination rate of *Arabidopsis thaliana* seeds post-drought treatment

	1/2MS		1/2MS+10%PEG6000		1/2MS+20%PEG6000		1/2MS+30%PEG6000	
	Col-0	*PAN*	Col-0	*PAN*	Col-0	*PAN*	Col-0	*PAN*
Total spotted seeds	26	20	29	21	32	25	27	16
Total germinated seeds	24	10	26	9	28	8	23	3
Germination rate	92%	50%	90%	43%	88%	32%	85%	19%

Table S4. List of the *TGA* genes ID identified in this study

ID	Gene Name	Species(Num.)	ID	Gene Name	Species(Num.)
AT5G65210	AtTGA1	*Arabidopsis thaliana*(10)	fgenesh2_kg.6__632__AT5G06839.2	AIyTGA1	*Arabidopsis lyrata*(10)
AT5G06950	AtTGA2		fgenesh2_kg.1__2395__AT1G22070.1	AIyTGA2	
AT1G22070	AtTGA3		fgenesh2_kg.6__963__AT5G10030.1	AIyTGA3	
AT5G10030	AtTGA4		Al_scaffold_0008_3070	AIyTGA4	
AT5G06960	AtTGA5		fgenesh2_kg.6__648__AT5G06960.1	AIyTGA5	
AT3G12250	AtTGA6		fgenesh2_kg.6__644__AT5G06950.1	AIyTGA6	
AT1G77920	AtTGA7		fgenesh1_pm.C_scaffold_1000665	AIyTGA7	
AT1G68640	AtTGA8		fgenesh2_kg.2__1120__AT1G68640.1	AIyTGA8	
AT1G08320	AtTGA9		Al_scaffold_0002_2497	AIyTGA9	
AT5G06839	AtTGA10		fgenesh2_kg.3__1288__AT3G12250.4	AIyTGA10	
g18268.t1	ArhTGA1	*Arabidopsis halleri*(13)	Csa13g011780.1	CsaTGA1	*Camelina sativa*(33)
g05806.t1	ArhTGA2		Csa20g009310.1	CsaTGA2	
g14725.t1	ArhTGA3		Csa18g038960.1	CsaTGA3	
g05806.t2	ArhTGA4		Csa16g028670.2	CsaTGA4	
g19179.t1	ArhTGA5		Csa16g028670.1	CsaTGA5	
g15619.t2	ArhTGA6		Csa08g057500.1	CsaTGA6	
g15634.t1	ArhTGA7		Csa17g028970.1	CsaTGA7	
g15619.t1	ArhTGA8		Csa09g084700.1	CsaTGA8	
g07444.t1	ArhTGA9		Csa20g015290.1	CsaTGA9	
g15633.t1	ArhTGA10		Csa03g011780.1	CsaTGA10	
g13342.t1	ArhTGA11		Csa02g073440.1	CsaTGA11	
g01352.t1	ArhTGA12		Csa19g016500.1	CsaTGA12	
g13342.t2	ArhTGA13		Csa08g057510.1	CsaTGA13	

cds.BjuA02g42590S	BjuTGA1		Csa08g054570.1	CsaTGA14	
cds.BjuA06g14910S	BjuTGA2		Csa16g042150.1	CsaTGA15	
cds.BjuA09g63330S	BjuTGA3		Csa07g034330.1	CsaTGA16	
cds.BjuA10g24030S	BjuTGA4		Csa14g009790.1	CsaTGA17	
cds.BjuA03g26260S	BjuTGA5		Csa14g027310.1	CsaTGA18	
cds.BjuA10g25800S	BjuTGA6		Csa07g034330.2	CsaTGA19	
cds.BjuB07g36040S	BjuTGA7		Csa14g027310.2	CsaTGA20	
cds.BjuB03g42580S	BjuTGA8		Csa03g025960.1	CsaTGA21	
cds.BjuB06g15300S	BjuTGA9		Csa13g008860.1	CsaTGA22	
cds.BjuB07g47010S	BjuTGA10		Csa01g013360.1	CsaTGA23	
cds.BjuA06g37280S	BjuTGA11		Csa05g085100.1	CsaTGA24	
cds.BjuB03g11930S	BjuTGA12		Csa17g011780.1	CsaTGA25	
cds.BjuA01g05270S	BjuTGA13	*Brassica juncea*(30)	Csa07g050580.1	CsaTGA26	
cds.BjuA07g23400S	BjuTGA14		Csa20g009180.1	CsaTGA27	
cds.BjuB02g44340S	BjuTGA15		Csa08g057630.1	CsaTGA28	
cds.BjuA08g23640S	BjuTGA16		Csa08g057630.2	CsaTGA29	
cds.BjuB07g21140S	BjuTGA17		Csa13g008990.1	CsaTGA30	
cds.BjuB05g40210S	BjuTGA18		Csa11g102430.1	CsaTGA31	
cds.BjuA07g36570S	BjuTGA19		Csa15g016140.1	CsaTGA32	
cds.BjuB03g20790S	BjuTGA20		Csa20g009280.1	CsaTGA33	
cds.BjuB04g23440S	BjuTGA21		Bo9g175780.1	BoTGA1	
cds.BjuB04g53260S	BjuTGA22		Bo3g004060.1	BoTGA2	
cds.BjuB02g03580S	BjuTGA23		Bo8g071300.1	BoTGA3	*Brassica oleracea*(19)
cds.BjuB02g44280S	BjuTGA24		Bo2g009670.1	BoTGA4	
cds.BjuB05g38710S	BjuTGA25		Bo6g121590.1	BoTGA5	

cds.BjuA02g00790S	BjuTGA26		Bo5g133550.1	BoTGA6	
cds.BjuA09g22940S	BjuTGA27		Bo3g099410.1	BoTGA7	
cds.BjuB02g42400S	BjuTGA28		Bo9g173550.1	BoTGA8	
cds.BjuB01g07050S	BjuTGA29		Bo6g081910.1	BoTGA9	
cds.BjuA02g40080S	BjuTGA30		Bo9g175710.1	BoTGA10	
			Bo5g008940.1	BoTGA11	
			Bo1g139470.1	BoTGA12	
			Bo9g018570.1	BoTGA13	
			Bo8g112540.1	BoTGA14	
			Bo6g107140.1	BoTGA15	
			Bo2g007460.1	BoTGA16	
			Bo00615s190.1	BoTGA17	
			Bo3g064830.1	BoTGA18	
			Bo5g035170.1	BoTGA19	

Table S5 Collinear gene pairs identified in *B. napus* and other plants

Arabidopsis thaliana	*B. napus*	*Arabidopsis lyrata*	*B. napus*	*Arabidopsis halleri*	*B. napus*	*Brassica juncea*	*B. napus*	*Brassica oleracea*	*B. napus*	*Camelina sativa*	*B. napus*
AtTGA9	BnTGA20	AIyTGA2	BnTGA26	ArhTGA9	BnTGA36	BjuTGA13	BnTGA31	BoTGA12	BnTGA31	CsaTGA23	BnTGA31
AtTGA8	BnTGA18	AIyTGA2	BnTGA4	ArhTGA9	BnTGA2	BjuTGA13	BnTGA28	BoTGA12	BnTGA28	CsaTGA23	BnTGA28
AtTGA7	BnTGA26	AIyTGA2	BnTGA35	ArhTGA9	BnTGA23	BjuTGA13	BnTGA12	BoTGA12	BnTGA12	CsaTGA23	BnTGA12
AtTGA3	BnTGA26	AIyTGA7	BnTGA15	ArhTGA9	BnTGA1	BjuTGA13	BnTGA11	BoTGA12	BnTGA19	CsaTGA23	BnTGA19
AtTGA7	BnTGA4	AIyTGA2	BnTGA24	ArhTGA9	BnTGA3	BjuTGA13	BnTGA6	BoTGA12	BnTGA11	CsaTGA23	BnTGA11
AtTGA3	BnTGA4	AIyTGA2	BnTGA34	ArhTGA9	BnTGA16	BjuTGA13	BnTGA13	BoTGA12	BnTGA6	CsaTGA23	BnTGA6
AtTGA3	BnTGA35	AIyTGA2	BnTGA14	ArhTGA9	BnTGA25	BjuTGA13	BnTGA32	BoTGA12	BnTGA13	CsaTGA23	BnTGA13
AtTGA7	BnTGA35	AIyTGA2	BnTGA17	ArhTGA9	BnTGA5	BjuTGA13	BnTGA29	BoTGA12	BnTGA32	CsaTGA23	BnTGA32
AtTGA9	BnTGA15	AIyTGA2	BnTGA21	ArhTGA11	BnTGA26	BjuTGA30	BnTGA36	BoTGA12	BnTGA29	CsaTGA23	BnTGA29
AtTGA3	BnTGA24	AIyTGA7	BnTGA9	ArhTGA11	BnTGA35	BjuTGA1	BnTGA12	BoTGA12	BnTGA38	CsaTGA23	BnTGA38
AtTGA7	BnTGA24	AIyTGA9	BnTGA26	ArhTGA11	BnTGA14	BjuTGA30	BnTGA2	BoTGA4	BnTGA36	CsaTGA11	BnTGA36
AtTGA9	BnTGA27	AIyTGA8	BnTGA18	ArhTGA11	BnTGA21	BjuTGA26	BnTGA2	BoTGA16	BnTGA28	CsaTGA11	BnTGA2
AtTGA3	BnTGA34	AIyTGA9	BnTGA4	ArhTGA4	BnTGA31	BjuTGA30	BnTGA23	BoTGA16	BnTGA12	CsaTGA11	BnTGA23
AtTGA7	BnTGA34	AIyTGA9	BnTGA35	ArhTGA4	BnTGA28	BjuTGA1	BnTGA19	BoTGA4	BnTGA23	CsaTGA11	BnTGA1
AtTGA7	BnTGA14	AIyTGA9	BnTGA24	ArhTGA4	BnTGA12	BjuTGA1	BnTGA11	BoTGA16	BnTGA19	CsaTGA11	BnTGA3
AtTGA3	BnTGA14	AIyTGA9	BnTGA34	ArhTGA4	BnTGA11	BjuTGA26	BnTGA1	BoTGA16	BnTGA11	CsaTGA11	BnTGA7
AtTGA8	BnTGA39	AIyTGA9	BnTGA14	ArhTGA4	BnTGA6	BjuTGA30	BnTGA7	BoTGA4	BnTGA7	CsaTGA11	BnTGA16
AtTGA7	BnTGA17	AIyTGA8	BnTGA39	ArhTGA4	BnTGA13	BjuTGA1	BnTGA6	BoTGA16	BnTGA6	CsaTGA11	BnTGA25
AtTGA3	BnTGA17	AIyTGA9	BnTGA17	ArhTGA4	BnTGA32	BjuTGA26	BnTGA3	BoTGA16	BnTGA32	CsaTGA11	BnTGA5
AtTGA3	BnTGA21	AIyTGA9	BnTGA21	ArhTGA4	BnTGA29	BjuTGA26	BnTGA16	BoTGA4	BnTGA5	CsaTGA10	BnTGA20
AtTGA7	BnTGA21	AIyTGA10	BnTGA31	ArhTGA4	BnTGA38	BjuTGA1	BnTGA32	BoTGA16	BnTGA29	CsaTGA21	BnTGA26
AtTGA9	BnTGA9	AIyTGA10	BnTGA28	ArhTGA1	BnTGA20	BjuTGA30	BnTGA5	BoTGA18	BnTGA31	CsaTGA21	BnTGA4
AtTGA6	BnTGA31	AIyTGA10	BnTGA12	ArhTGA1	BnTGA15	BjuTGA1	BnTGA29	BoTGA18	BnTGA28	CsaTGA21	BnTGA35
AtTGA6	BnTGA28	AIyTGA10	BnTGA11	ArhTGA1	BnTGA27	BjuTGA26	BnTGA25	BoTGA18	BnTGA12	CsaTGA10	BnTGA15
AtTGA6	BnTGA12	AIyTGA10	BnTGA6	ArhTGA3	BnTGA18	BjuTGA5	BnTGA31	BoTGA7	BnTGA2	CsaTGA21	BnTGA24
AtTGA6	BnTGA11	AIyTGA10	BnTGA13	ArhTGA3	BnTGA39	BjuTGA5	BnTGA28	BoTGA2	BnTGA30	CsaTGA10	BnTGA27
AtTGA6	BnTGA6	AIyTGA10	BnTGA32	ArhTGA8	BnTGA28	BjuTGA5	BnTGA12	BoTGA18	BnTGA11	CsaTGA21	BnTGA34
AtTGA6	BnTGA13	AIyTGA10	BnTGA29	ArhTGA10	BnTGA12	BjuTGA5	BnTGA11	BoTGA7	BnTGA1	CsaTGA21	BnTGA14
AtTGA6	BnTGA32	AIyTGA10	BnTGA38	ArhTGA8	BnTGA30	BjuTGA5	BnTGA6	BoTGA7	BnTGA3	CsaTGA21	BnTGA17
AtTGA6	BnTGA29	AIyTGA3	BnTGA36	ArhTGA10	BnTGA19	BjuTGA5	BnTGA13	BoTGA18	BnTGA6	CsaTGA21	BnTGA21
AtTGA6	BnTGA38	AIyTGA1	BnTGA28	ArhTGA10	BnTGA11	BjuTGA5	BnTGA32	BoTGA7	BnTGA16	CsaTGA24	BnTGA18
AtTGA4	BnTGA36	AIyTGA6	BnTGA12	ArhTGA10	BnTGA6	BjuTGA5	BnTGA37	BoTGA18	BnTGA13	CsaTGA24	BnTGA39
AtTGA2	BnTGA28	AIyTGA3	BnTGA29	ArhTGA10	BnTGA32	BjuTGA5	BnTGA38	BoTGA18	BnTGA32	CsaTGA26	BnTGA26
AtTGA2	BnTGA12	AIyTGA3	BnTGA23	ArhTGA8	BnTGA37	BjuTGA2	BnTGA2	BoTGA7	BnTGA25	CsaTGA16	BnTGA18
AtTGA1	BnTGA29	AIyTGA1	BnTGA30	ArhTGA10	BnTGA29	BjuTGA11	BnTGA20	BoTGA2	BnTGA37	CsaTGA26	BnTGA4
AtTGA4	BnTGA29	AIyTGA6	BnTGA19	ArhTGA12	BnTGA26	BjuTGA11	BnTGA15	BoTGA18	BnTGA37	CsaTGA26	BnTGA35
AtTGA1	BnTGA23	AIyTGA6	BnTGA11	ArhTGA12	BnTGA4	BjuTGA2	BnTGA1	BoTGA18	BnTGA38	CsaTGA26	BnTGA24
AtTGA4	BnTGA23	AIyTGA6	BnTGA6	ArhTGA12	BnTGA35	BjuTGA2	BnTGA3	BoTGA6	BnTGA31	CsaTGA26	BnTGA34

AtTGA10	BnTGA30	AIyTGA3	BnTGA7	ArhTGA12	BnTGA24	BjuTGA2	BnTGA16	BoTGA6	BnTGA28	CsaTGA26	BnTGA14
AtTGA2	BnTGA19	AIyTGA3	BnTGA16	ArhTGA12	BnTGA34	BjuTGA11	BnTGA27	BoTGA6	BnTGA12	CsaTGA16	BnTGA39
AtTGA2	BnTGA11	AIyTGA1	BnTGA13	ArhTGA12	BnTGA14	BjuTGA2	BnTGA25	BoTGA11	BnTGA20	CsaTGA26	BnTGA17
AtTGA1	BnTGA1	AIyTGA6	BnTGA32	ArhTGA12	BnTGA17	BjuTGA14	BnTGA4	BoTGA19	BnTGA26	CsaTGA26	BnTGA21
AtTGA1	BnTGA3	AIyTGA3	BnTGA5	ArhTGA12	BnTGA21	BjuTGA19	BnTGA26	BoTGA19	BnTGA4	CsaTGA14	BnTGA36
AtTGA1	BnTGA7	AIyTGA1	BnTGA37	ArhTGA5	BnTGA36	BjuTGA14	BnTGA26	BoTGA19	BnTGA35	CsaTGA6	BnTGA28
AtTGA2	BnTGA6	AIyTGA6	BnTGA29	ArhTGA5	BnTGA2	BjuTGA19	BnTGA4	BoTGA19	BnTGA24	CsaTGA6	BnTGA12
AtTGA4	BnTGA7	AIyTGA4	BnTGA29	ArhTGA5	BnTGA23	BjuTGA14	BnTGA35	BoTGA11	BnTGA15	CsaTGA14	BnTGA2
AtTGA4	BnTGA16	AIyTGA4	BnTGA23	ArhTGA5	BnTGA7	BjuTGA19	BnTGA35	BoTGA6	BnTGA11	CsaTGA14	BnTGA23
AtTGA1	BnTGA16	AIyTGA4	BnTGA1	ArhTGA5	BnTGA16	BjuTGA14	BnTGA24	BoTGA6	BnTGA6	CsaTGA6	BnTGA19
AtTGA2	BnTGA32	AIyTGA4	BnTGA37	ArhTGA5	BnTGA5	BjuTGA19	BnTGA24	BoTGA6	BnTGA13	CsaTGA28	BnTGA30
AtTGA1	BnTGA25	AIyTGA4	BnTGA7			BjuTGA19	BnTGA34	BoTGA6	BnTGA32	CsaTGA6	BnTGA11
AtTGA1	BnTGA5	AIyTGA4	BnTGA16			BjuTGA19	BnTGA14	BoTGA11	BnTGA27	CsaTGA14	BnTGA7
AtTGA4	BnTGA5	AIyTGA4	BnTGA25			BjuTGA14	BnTGA17	BoTGA19	BnTGA34	CsaTGA6	BnTGA6
AtTGA10	BnTGA37	AIyTGA4	BnTGA5			BjuTGA14	BnTGA14	BoTGA19	BnTGA14	CsaTGA14	BnTGA16
AtTGA2	BnTGA29					BjuTGA19	BnTGA17	BoTGA19	BnTGA17	CsaTGA6	BnTGA32
						BjuTGA14	BnTGA21	BoTGA19	BnTGA21	CsaTGA14	BnTGA5
						BjuTGA16	BnTGA26	BoTGA6	BnTGA38	CsaTGA6	BnTGA29
						BjuTGA16	BnTGA35	BoTGA5	BnTGA26	CsaTGA28	BnTGA37
						BjuTGA16	BnTGA24	BoTGA15	BnTGA18	CsaTGA14	BnTGA25
						BjuTGA16	BnTGA14	BoTGA5	BnTGA4	CsaTGA8	BnTGA26
						BjuTGA16	BnTGA21	BoTGA9	BnTGA26	CsaTGA8	BnTGA4
						BjuTGA3	BnTGA36	BoTGA5	BnTGA35	CsaTGA8	BnTGA35
						BjuTGA3	BnTGA2	BoTGA9	BnTGA24	CsaTGA8	BnTGA24
						BjuTGA27	BnTGA26	BoTGA5	BnTGA24	CsaTGA8	BnTGA34
						BjuTGA27	BnTGA4	BoTGA5	BnTGA34	CsaTGA8	BnTGA14
						BjuTGA27	BnTGA35	BoTGA5	BnTGA14	CsaTGA8	BnTGA17
						BjuTGA27	BnTGA24	BoTGA9	BnTGA17	CsaTGA8	BnTGA21
						BjuTGA3	BnTGA1	BoTGA15	BnTGA39	CsaTGA31	BnTGA2
						BjuTGA3	BnTGA3	BoTGA9	BnTGA14	CsaTGA31	BnTGA23
						BjuTGA3	BnTGA16	BoTGA5	BnTGA17	CsaTGA31	BnTGA1
						BjuTGA27	BnTGA34	BoTGA5	BnTGA21	CsaTGA31	BnTGA3
						BjuTGA27	BnTGA14	BoTGA14	BnTGA20	CsaTGA31	BnTGA16
						BjuTGA27	BnTGA17	BoTGA3	BnTGA26	CsaTGA31	BnTGA25
						BjuTGA3	BnTGA25	BoTGA3	BnTGA4	CsaTGA31	BnTGA5
						BjuTGA4	BnTGA36	BoTGA3	BnTGA35	CsaTGA1	BnTGA36
						BjuTGA6	BnTGA28	BoTGA14	BnTGA15	CsaTGA30	BnTGA28
						BjuTGA4	BnTGA23	BoTGA3	BnTGA24	CsaTGA30	BnTGA12
						BjuTGA6	BnTGA19	BoTGA14	BnTGA27	CsaTGA1	BnTGA2
						BjuTGA6	BnTGA11	BoTGA3	BnTGA34	CsaTGA1	BnTGA23
						BjuTGA4	BnTGA7	BoTGA3	BnTGA14	CsaTGA30	BnTGA19

						BjuTGA6	BnTGA6	BoTGA3	BnTGA17	CsaTGA22	BnTGA30
						BjuTGA4	BnTGA5	BoTGA3	BnTGA21	CsaTGA30	BnTGA11
						BjuTGA6	BnTGA29	BoTGA13	BnTGA36	CsaTGA1	BnTGA7
						BjuTGA29	BnTGA31	BoTGA8	BnTGA36	CsaTGA30	BnTGA6
						BjuTGA29	BnTGA28	BoTGA1	BnTGA28	CsaTGA1	BnTGA16
						BjuTGA29	BnTGA12	BoTGA10	BnTGA12	CsaTGA30	BnTGA32
						BjuTGA29	BnTGA19	BoTGA13	BnTGA2	CsaTGA1	BnTGA5
						BjuTGA29	BnTGA11	BoTGA8	BnTGA2	CsaTGA30	BnTGA29
						BjuTGA29	BnTGA6	BoTGA8	BnTGA23	CsaTGA22	BnTGA37
						BjuTGA29	BnTGA13	BoTGA10	BnTGA19	CsaTGA17	BnTGA20
						BjuTGA29	BnTGA32	BoTGA1	BnTGA30	CsaTGA18	BnTGA26
						BjuTGA29	BnTGA29	BoTGA13	BnTGA1	CsaTGA18	BnTGA4
						BjuTGA29	BnTGA38	BoTGA10	BnTGA11	CsaTGA18	BnTGA35
						BjuTGA28	BnTGA36	BoTGA8	BnTGA7	CsaTGA17	BnTGA15
						BjuTGA24	BnTGA28	BoTGA10	BnTGA6	CsaTGA18	BnTGA24
						BjuTGA23	BnTGA20	BoTGA13	BnTGA3	CsaTGA17	BnTGA27
						BjuTGA23	BnTGA15	BoTGA13	BnTGA16	CsaTGA18	BnTGA34
						BjuTGA28	BnTGA23	BoTGA8	BnTGA16	CsaTGA18	BnTGA14
						BjuTGA24	BnTGA30	BoTGA8	BnTGA5	CsaTGA18	BnTGA17
						BjuTGA24	BnTGA11	BoTGA10	BnTGA29	CsaTGA18	BnTGA21
						BjuTGA28	BnTGA7	BoTGA1	BnTGA37	CsaTGA17	BnTGA9
						BjuTGA24	BnTGA6	BoTGA13	BnTGA25	CsaTGA32	BnTGA31
						BjuTGA28	BnTGA16	BoTGA17	BnTGA2	CsaTGA32	BnTGA28
						BjuTGA23	BnTGA27	BoTGA17	BnTGA1	CsaTGA32	BnTGA12
						BjuTGA23	BnTGA9	BoTGA17	BnTGA3	CsaTGA32	BnTGA11
						BjuTGA28	BnTGA5	BoTGA17	BnTGA16	CsaTGA32	BnTGA6
						BjuTGA24	BnTGA29	BoTGA17	BnTGA25	CsaTGA32	BnTGA13
						BjuTGA12	BnTGA4			CsaTGA32	BnTGA32
						BjuTGA20	BnTGA18			CsaTGA32	BnTGA29
						BjuTGA12	BnTGA26			CsaTGA32	BnTGA38
						BjuTGA12	BnTGA35			CsaTGA15	BnTGA26
						BjuTGA8	BnTGA35			CsaTGA5	BnTGA18
						BjuTGA12	BnTGA24			CsaTGA15	BnTGA4
						BjuTGA8	BnTGA24			CsaTGA15	BnTGA35
						BjuTGA12	BnTGA34			CsaTGA15	BnTGA24
						BjuTGA12	BnTGA17			CsaTGA15	BnTGA34
						BjuTGA12	BnTGA14			CsaTGA15	BnTGA14
						BjuTGA20	BnTGA39			CsaTGA5	BnTGA39
						BjuTGA8	BnTGA21			CsaTGA15	BnTGA17
						BjuTGA12	BnTGA21			CsaTGA15	BnTGA21
						BjuTGA22	BnTGA2			CsaTGA25	BnTGA20

						BjuTGA21	BnTGA4			CsaTGA7	BnTGA26
						BjuTGA21	BnTGA26			CsaTGA7	BnTGA4
						BjuTGA21	BnTGA35			CsaTGA7	BnTGA35
						BjuTGA21	BnTGA24			CsaTGA25	BnTGA15
						BjuTGA22	BnTGA1			CsaTGA7	BnTGA24
						BjuTGA22	BnTGA3			CsaTGA25	BnTGA27
						BjuTGA22	BnTGA16			CsaTGA7	BnTGA34
						BjuTGA21	BnTGA34			CsaTGA7	BnTGA14
						BjuTGA21	BnTGA17			CsaTGA7	BnTGA17
						BjuTGA21	BnTGA14			CsaTGA7	BnTGA21
						BjuTGA22	BnTGA25			CsaTGA3	BnTGA2
						BjuTGA22	BnTGA5			CsaTGA3	BnTGA23
						BjuTGA18	BnTGA36			CsaTGA3	BnTGA1
						BjuTGA25	BnTGA28			CsaTGA3	BnTGA3
						BjuTGA25	BnTGA12			CsaTGA3	BnTGA16
						BjuTGA18	BnTGA23			CsaTGA3	BnTGA25
						BjuTGA25	BnTGA19			CsaTGA3	BnTGA5
						BjuTGA25	BnTGA11			CsaTGA12	BnTGA31
						BjuTGA25	BnTGA6			CsaTGA12	BnTGA28
						BjuTGA18	BnTGA7			CsaTGA12	BnTGA12
						BjuTGA18	BnTGA5			CsaTGA12	BnTGA11
						BjuTGA25	BnTGA29			CsaTGA12	BnTGA6
						BjuTGA9	BnTGA4			CsaTGA12	BnTGA13
						BjuTGA9	BnTGA26			CsaTGA12	BnTGA32
						BjuTGA9	BnTGA35			CsaTGA12	BnTGA29
						BjuTGA9	BnTGA17			CsaTGA12	BnTGA38
						BjuTGA9	BnTGA14			CsaTGA9	BnTGA36
						BjuTGA10	BnTGA31			CsaTGA33	BnTGA28
						BjuTGA17	BnTGA31			CsaTGA33	BnTGA12
						BjuTGA7	BnTGA36			CsaTGA9	BnTGA2
						BjuTGA17	BnTGA28			CsaTGA9	BnTGA23
						BjuTGA10	BnTGA28			CsaTGA33	BnTGA19
						BjuTGA10	BnTGA12			CsaTGA27	BnTGA30
						BjuTGA17	BnTGA12			CsaTGA33	BnTGA11
						BjuTGA7	BnTGA2			CsaTGA9	BnTGA7
						BjuTGA7	BnTGA23			CsaTGA33	BnTGA6
						BjuTGA7	BnTGA1			CsaTGA9	BnTGA16
						BjuTGA17	BnTGA11			CsaTGA33	BnTGA32
						BjuTGA10	BnTGA11			CsaTGA9	BnTGA25
						BjuTGA7	BnTGA3			CsaTGA9	BnTGA5
						BjuTGA10	BnTGA6			CsaTGA33	BnTGA29

						BjuTGA17	BnTGA13			CsaTGA27	BnTGA37
						BjuTGA7	BnTGA16				
						BjuTGA10	BnTGA13				
						BjuTGA10	BnTGA32				
						BjuTGA17	BnTGA32				
						BjuTGA7	BnTGA25				
						BjuTGA10	BnTGA29				
						BjuTGA7	BnTGA5				
						BjuTGA10	BnTGA38				

Table S6 Statistical analysis of *cis*-acting elements of TGA gene family

Founctions	Elements	BnTGA1	BnTGA2	BnTGA3	BnTGA4	BnTGA5	BnTGA6	BnTGA7	BnTGA8	BnTGA9	BnTGA10	BnTGA11	BnTGA12	BnTGA13	BnTGA14	BnTGA15	BnTGA16	BnTGA17	BnTGA18	BnTGA19	BnTGA20	BnTGA21	BnTGA22	BnTGA23	BnTGA24	BnTGA25	BnTGA26	BnTGA27	BnTGA28	BnTGA29	BnTGA30	BnTGA31	BnTGA32	BnTGA33	BnTGA34	BnTGA35	BnTGA36	BnTGA37	BnTGA38	BnTGA39	Summary
wound-responsive element	WUN-motif	1		1	1			3	1	1	1		1				2	2	2		1				2	2		1									1			2	25
light responsive	AE-box	1	2	1	1	1				1	1				1	2	1	1	1		1			1			1	1						1	2						21
	AT-rich element																				1																				1
	CCAAT-box		1			1							1							1		1	1	1			1					1	2						1		12
	AT1-motif				2				1																																3
	Box II																			1										1						1					3
	chs-CMA1a		1								2															1															4
	chs-CMA2a																													1					1			1			3
	Gap-box				1					1			1																	1		1									5
	GA-motif	1	1	1				1									1		2			1															1	1		5	15
	GATA-motif	1	2	2	1	1			1	1				1		2	1					2			2				1	1		1	1						1		22
	Sp1																															1		2					1		4
	GT1-motif	4	1	3	1		2	5		2	1		2	1	3	1		3	1	3		1					1	1				2	3				6		1	1	49
	I-box	2	2	2	2		1				1	2				1	2	2								1					1										19
	ACE						1					1											1												1						4
	TCCC-motif									1				1		1					1				1	1		1													7
	TCT-motif		1		1	1	2	1	2		1	1	1	1	1		1	1	1	2	1	1		1	1		2	1	1	1	1	1		1		1	2	1	1	2	37
	ATCT-motif					1														1		2							1						1						6
	Box 4	3	3	5	1	2	2	1	6	4	2	1	2	1	1	3	1	1	4	2	4	2	1	3		3		2	1	1	1	1	2	6	4	1	1		1	4	83
	G-Box	5	4	5	2	4	1	1	3	5	1	1	6	7	3	1	5	3	1	4	2	1	4	4		3	2	2	6	4		5	6		3	3	2		4		113
	MRE		1			1		1	1		1							1				1			1	1									1					1	11
drought-inducibility	MBS	3	1	2		1	1				1	1	1	1	2					1			2	1		3			1	3		4	2			1			2		34
anoxic specific inducibility	GC-motif													1															1												2
MeJA-responsive	CGTCA-motif	4	4	4		2	1	4	1	1	1	1		1	2		2			1	1	3	2	2		2	2	1	1	2	2	1		2	1	3	4	2	1		61
	TGACG-	4	4	4		2	1	4	1	1	1	1		1	2		3			1	1	3	2	2		2	2		1	2	2	1		2	1	3	4	2	1		61

	motif																																							
anaerobic induction	ARE	6		4		2	1	3	2	1	2	1	4	6	2		3		4	1	1	3	3	2	5	2	2	1	6	1	2	2	5	1	3	3	2	2	4	4	96
abscisic acid responsive	ABRE	2	4	2	2	3	1		3	4	2	2	3	4	1	1	3	3	1	3	2	2	8	3			1	2	4	3		5	6		3	4		1	4	1	93
	SARE																																	1							1
salicylic acid responsive	TCA-element	2		2	1		1		1		1	1	1				1	1	3							1	2				1	3	2	4	2				3	4	37
low-temperature responsive	LTR				1					1	2				2			3		2			1			1			1	2		1	1		1	3		1	1	1	25
gibberellin-responsive	GARE-motif				1													1					3								1		1					1			8
	P-box		1		1	1		1						2			1	3					1	1					2		1	1	1					1	1		19
	TATC-box						1					1	1		1						1						2												2		9
defense and stress responsiveness	TC-rich repeats		2		2		2			2						1		3	2			1	1			2							1	1	1	2				2	25
auxin-responsive element	AuxRR-core						1					1																													2
	TGA-element	1	1	1	1		2	1	1		1	1	3	2					1			1			1	1		1	2			2	1			1			2		28
	Summary	40	36	39	22	23	21	26	24	26	22	16	27	30	21	13	27	28	23	23	17	25	30	21	13	26	18	14	29	23	12	33	34	21	25	26	23	13	31	27	948

Table S7 The primer sequences used in qRT-PCR

No.	Primer name	Forward Primer (5'to3')	Reverse Primer (5'to3')
1	Actin	AGCGACCACCTTAATCTTC	GGTTTTGTTCCAGCCATC
2	BnTGA14	CCTCAAATGGCTGCAGCG	CGCCATTTGCTGCAACGT
3	BnTGA23	ACCACAAGACAAGCGGCT	TGGTTCACGTTGCCGAGT
4	BnTGA17	GGGCAGCATCGAGTTGGA	AGCGTCCCTTTGCCATCG
